# Blended CBT versus face-to-face CBT: a randomised non-inferiority trial

**DOI:** 10.1186/s12888-016-1140-y

**Published:** 2016-12-05

**Authors:** Kim Mathiasen, Tonny E. Andersen, Heleen Riper, Annet A. M. Kleiboer, Kirsten K. Roessler

**Affiliations:** 1 Department of Psychology, University of Southern Denmark, Campusvej 55, Odense M, 5230 Denmark; 2 Centre for Telepsychiatry, Mental Health Services of Southern Denmark, Heden 11, Odense C, 5000 Denmark; 3 Department of Clinical Psychology, VU University of Amsterdam, Van der Boechorststraat 1, Amsterdam, 1081HV The Netherlands

## Abstract

**Background:**

Internet based cognitive behavioural therapy (iCBT) has been demonstrated to be cost- and clinically effective. There is a need, however, for increased therapist contact for some patient groups. Combining iCBT with traditional face-to-face (ftf) consultations in a blended format (B-CBT) may produce a new treatment format with multiple benefits from both traditional CBT and iCBT such as individual adaptation, lower costs than traditional therapy, wide geographical and temporal availability, and possibly lower threshold to implementation. The primary aim of the present study is to compare directly the clinical effectiveness of B-CBT with face-to-face CBT for adult major depressive disorder.

**Methods/Design:**

The study is designed as a two arm randomised controlled non-inferiority trial comparing blended CBT for adult depression with treatment as usual (TAU). In the blended condition six sessions of ftf CBT is alternated with six to eight online modules (NoDep). TAU is defined as 12 sessions of ftf CBT. The primary outcome is symptomatic change of depressive symptoms on the patient-health questionnaire (PHQ-9). Additionally, the study will include an economic evaluation. All participants must be 18 years of age or older and meet the diagnostic criteria for major depressive disorder according to the Diagnostic and Statistical Manual of Mental disorders 4th edition. Participants are randomised on an individual level by a researcher not involved in the project. The primary outcome is analysed by regressing the three-month follow-up PHQ-9 data on the baseline PHQ-9 score and a treatment group indicator using ancova. A sample size of 130 in two balanced groups will yield a power of at least 80% to detect standardised mean differences above 0.5 on a normally distributed variable.

**Discussion:**

This study design will compare B-CBT and ftf CBT in a concise and direct manner with only a minimal of the variance explained by differences in therapeutic content. On the other hand, while situated in routine care, ecological validity is somewhat compromised by the controlled manner in which the study is conducted.

**Trial registration:**

ClinicalTrials.gov NCT02796573. Registered June 1st 2016. Currently recruiting participants.

## Background

Depression is a prevalent and disabling disorder with a high risk of relapse and large individual and societal costs [1–6]. Effective treatments exist [7], but there is a large gap between the need for and use of treatments [8]. Evidence based psychotherapies such as CBT, may help to bridge this gap. The reach of face-to-face (ftf) CBT is limited [9] however, which has led researchers to explore alternative modes of delivery of CBT such as Internet based guided self-help (iCBT) [10–16]. In spite of the evidence for the effect of iCBT, there is a need for increased therapist contact among some patient groups to overcome some of the shortcomings of iCBT [17–20].

Combining iCBT with traditional face-to-face consultations in a blended CBT format (B-CBT) may alleviate some of the difficulties associated with iCBT (such as lack of clinician contact), while still preserving some of the advantages of iCBT and face-to-face CBT alike. In B-CBT, online modules and face-to-face consultations are combined in one coherent treatment manual. This format could hold multiple benefits. Firstly, by providing face-to-face sessions, the therapist can individualise the therapy by taking the personal profile of the patient, the disorder, and possible co-morbidity into account. Secondly, since B-CBT only provides half the number of sessions as traditional face-to-face CBT, the capacity of the treating clinician is increased compared to traditional CBT. Third, the burden and cost of travel by the patient can be reduced. Fourth, the online modules are available at the time and place needed by the patients - and they can be re-viewed multiple times. Fifth, the inherently structured format of the online modules secures the same level of psycho-education and exercises to be delivered to all patients. Finally, one of the principal barriers for the uptake of iCBT seems to be skepticism from clinicians towards leaving the majority of therapy to a computer [21], a barrier possibly alleviated by the blended format in B-CBT.

Few studies have investigated the use of blended care combining internet based psychotherapeutic modules and face-to-face sessions into one coherent treatment manual [22–26]. Generally, however, they do indicate positive outcomes. In a randomised controlled trial conducted in primary care in Tromsø, Norway clinical psychologists delivered 30 min sessions following each online module [24]. They were able to document a significant difference with a moderate to large effect size (d = 0.65) on depressive symptoms (BDI-II) favouring blended care over waiting list. The intervention predominantly received positive evaluations suggesting acceptability and satisfaction with the treatment. Additionally, a qualitative study found that the face-to-face consultations increased motivation to persist with the iCBT programme [27]. Another recent example is the development and initial evaluation of a programme for B-CBT in The Netherlands. This was tested in an outpatient clinic of a specialised mental health care centre in Amsterdam. The study was designed as a feasibility study and included only nine patients. However, the patients perceived the intervention as positive although the authors rightly note that no conclusion can be derived from such a small sample [26].

## Aims and hypotheses

The primary aim of the present study is to compare the clinical effectiveness of blended cognitive behavioural therapy (B-CBT) for major depressive disorder (MDD) in adults with treatment as usual (TAU) defined as 12 sessions of face-to-face CBT. It is hypothesised that B-CBT will be no less clinically effective than TAU, and that it will be acceptable and satisfactory to patients and clinicians.

## Methods

### Design

The study is designed as a two arm randomised controlled non-inferiority trial comparing B-CBT for adult depression to treatment as usual (TAU) with an economic evaluation alongside. The present trial is part of the national project ENTER located and coordinated from the Center for Telepsychiatry and is additionally affiliated with the EU-study E-COMPARED [28].

### Participants

#### Inclusion criteria

All participants must be 18 years of age or older and meet the diagnostic criteria for major depressive disorder according to the Diagnostic and Statistical Manual of Mental disorders 4th edition (DSM-IV) [29]. Additionally, the participants must score ‘5’ or higher on the Patient Health Questionnaire-9 (PHQ-9) [30].

#### Exclusion criteria

Patients will be excluded in case of current high risk of suicide or if they suffer from a co-morbid substance dependence, bipolar affective disorder, psychotic illness, or obsessive compulsive disorder. Additionally, participants will be excluded if they currently receive psychological treatment for depression in primary or specialised mental health care. Finally, they will be excluded if they are unable to comprehend Danish or if they do not have access to a PC and Internet connection.

### Recruitment

Participants are recruited from the Centre for Telepsychiatry in specialised mental health care at the Mental Health Services of the Region of Southern Denmark, where they are referred to the study by clinicians. Initially, however, patients are self-referred to the Centre for Telepsychiatry.

The diagnosis will be confirmed by telephone administered interviews using the International Neuropsychiatric Interview version 5.0 (M.I.N.I.) [31] administered via telephone by trained masters students in psychology. Additionally, the participants are asked for permission from the research team to obtain the results of the PHQ-9 questionnaire from their application form to minimise the burden on the patients. Patients are asked to keep eventual antidepressant medication stable during treatment. If changes should occur, they are asked to report it.

### Randomisation and blinding

An independent researcher who is not involved in the trial performs the randomisation (centrally from Amsterdam) at an individual level, stratified by country after eligibility and baseline measurement. A computerised random number generator (Random Allocation Software) will be applied with an allocation ratio of 1:1. Block randomisation will be used with variable block sizes that vary between eight and fourteen allocations per block.

The assessors for eligibility will be blinded to group allocation. During treatment, blinding of participants and clinicians are naturally impossible. Researchers and statisticians involved in the analyses will be blinded to group allocations. The groups will be numbered in the dataset without revealing which treatment was given. The questionnaires administered solely to the B-CBT group will be analysed in a separate dataset.

### Interventions

#### Blended treatment (B-CBT)

B-CBT combines (blends) six individual face-to-face CBT sessions with six to eight online CBT modules delivered through an Internet based treatment programme. The face-to-face consultations will be provided by a psychologist at the Centre for Tele-Psychiatry.

The programme is a recently developed iCBT programme (NoDep) based on CBT for depression which includes six mandatory modules and two optional ones. The core components of the mandatory modules are: psycho-education, cognitive restructuring, behavioural activation, behaviour experiments, and relapse prevention. The optional modules comprise: coping with rumination, and restructuring of core beliefs. The decision whether to add any optional modules are taken jointly by the patient and the psychologist based on patient needs, motivation, and possible time constraints. See Table 1 for an overview of the intervention. All modules are delivered via multimedia elements including video, audio, interactive exercises, calendar, and pdf summaries. The program has a build in workflow predetermining the order in which the modules are presented. All data is stored in Europe and is encrypted during storage and transmission.

**Table 1:** Interventions

Session number	Format of delivery	Content	Example of exercise
	B-CBT		
1	F2F	Introduction and psychoeducation about depression and the treatment	Find a helper
2	Online module	Introduction to the programme, psychoeducation about depression, and goals for the treatment	Problem- goal list
3	F2F	Idiosyncratic model of the disorder	Cognitive case formulation
4	Online module	Psychoeducation about behaviour in depression	Activity registration
5	F2F	Accordance between personal values and behaviour. Introduction to cognitive restructuring.	Simple exercise for cognitive restructuring
6	Online module	Changing behaviour based on activity registration and personal values	Activity planning
7	F2F	Psychoeducation about negative automatic thoughts and cognitive restructuring.	Cognitive restructuring exercise
8	Online module	Psychoeducation about negative automatic thoughts and cognitive restructuring.	Cognitive restructuring exercise
9	F2F	Psychoeducation about behavioural experiments. Decision is made whether to include one or both of the extra modules	Behavioural experiment
10	Online module (A, B)	Behavioural experiments. (A: psychoeducation about core beliefs, B: coping with rumination)	Behavioural experiment (A: challenge core beliefs, B: test three techniques for coping with rumination)
11	F2F	Summing up, relapse prevention	Continuation of preferred exercises
12	Online module	Summing up, relapse prevention	Personal relapse prevention plan
	TAU		
1	F2F	Introduction and psychoeducation about depression and the treatment	Find a helper
2	F2F	Psychoeducation and goals for the treatment	Problem- goallist
3	F2F	Idiosyncratic model of the disorder	Cognitive case formulation
4	F2F	Psychoeducation about behaviour in depression	Activity registration
5	F2F	Accordance between personal values and behaviour. Introduction to cognitive restructuring.	Simple exercise for cognitive restructuring
6	F2F	Changing behaviour based on activity registration and personal values	Activity planning
7	F2F	Psychoeducation about negative automatic thoughts and cognitive restructuring.	Cognitive restructuring exercise
8	F2F	Psychoeducation about negative automatic thoughts and cognitive restructuring.	Cognitive restructuring exercise
9	F2F	Psychoeducation about behavioural experiments.	Behavioural experiment
10	F2F	Psychoeducation about core beliefs or continue working on behavioural experiments	Challenge core beliefs or behavioural experiment
11	F2F	Psychoeducation about rumination or beginning of relapse prevention	Test three techniques to cope with rumination or start personal relapse prevention plan and continuation of preferred exercise
12	F2F	Summing up, relapse prevention	Personal relapse prevention plan

For technical support to the participants, the existing procedures at Internetpsykiatrien are used, which consists of two levels: the first is handled by the clinicians, the second goes through an error report system to the company providing the software (Context Consulting).

### Treatment as usual (TAU)

TAU will be defined as 12 sessions of face-to-face CBT including the same core components as the B-CBT condition. Additionally, interventions on core beliefs and rumination can be included according to the same criteria as in the B-CBT condition. See Table 1 for an overview of the intervention.

Patients in either condition is monitored weekly for symptoms of depression including suicidal ideation and -intent. Should any participant deteriorate or show signs of suicidal intent, an assessment interview will be conducted and the patient will be discontinued if necessary and referred to relevant treatment.

### Adherence

Participants will receive automated sms and/or e-mail reminders of homework assignments and questionnaires. In case a patient is inactive for more than two days without prior agreement with the treating psychologist, the patient is contacted via e-mail and telephone. Additionally, a computer is setup at the clinic, for participants to engage with the online programme in case they are for some reason not able to do so at home. This includes lack of adherence even if they do not suffer technical difficulties.

#### Treatment fidelity

**Fig. 1:**
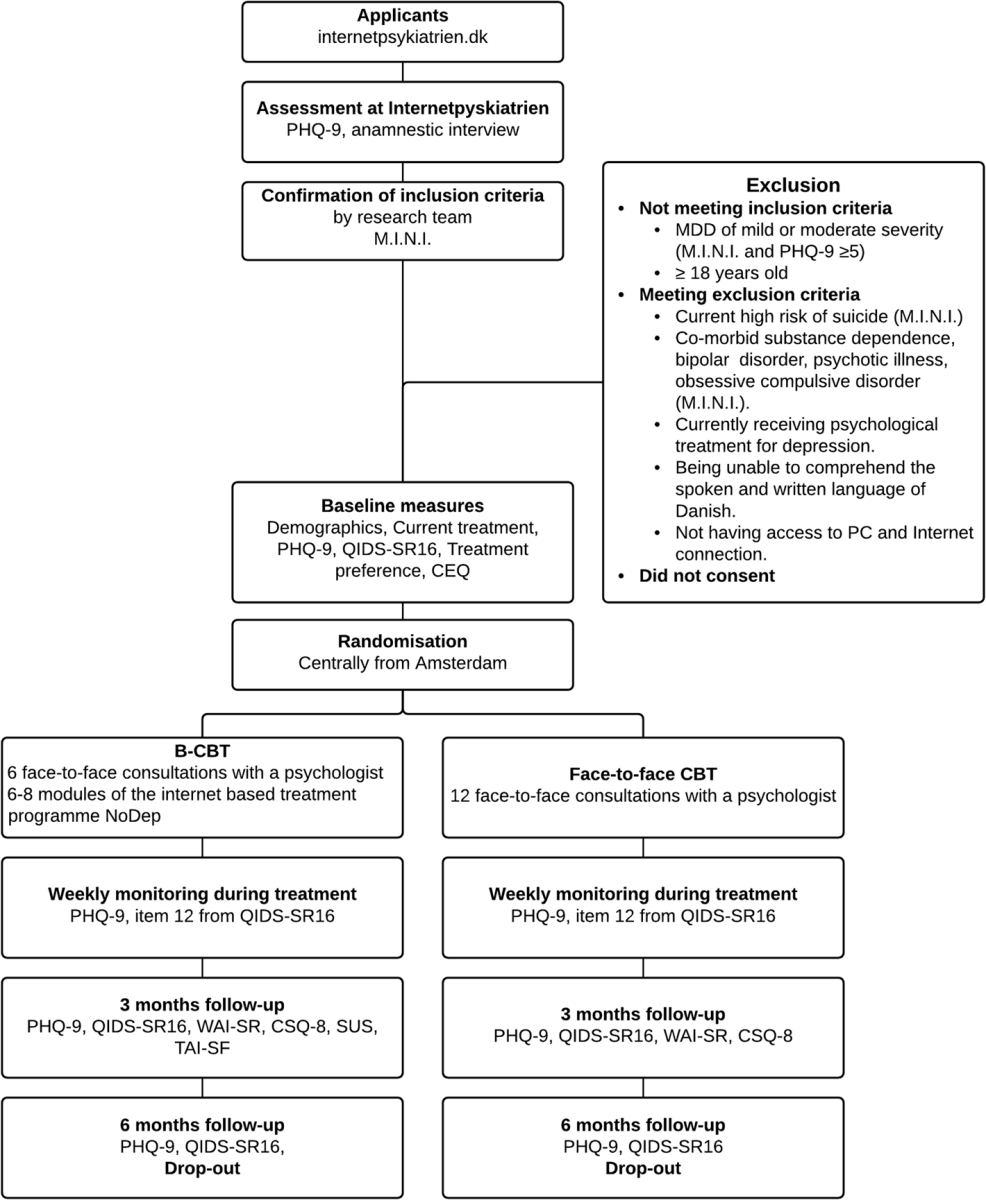
Patient flow diagramme

All therapists are clinical psychologists. To ensure treatment fidelity (1) detailed treatment manuals, one for each treatment condition, has been developed to guide therapists through the treatment, (2) regular supervision is provided by the research coordinator to prevent drift, (3) therapists will register the number of sessions, the frequency of the sessions, the main strategies used in each sessions and the length of each contact, and finally (4) all sessions will be audio recorded and approximately 5% of the sessions in each condition will be randomly selected and evaluated by an external CBT expert (Fig. 1).

### Outcome measurements

Baseline measures will be administered after consent has been granted prior to randomisation. The participants will be followed up three-, six-, and twelve months after baseline measurement. In case of drop-out, a short questionnaire regarding reasons for drop-out will be administered. All questionnaires for the participants will be administered online. A secure web application for building and managing online surveys (RedCap) will be used for all questionnaire packages except the weekly monitoring for the B-CBT group, which will be administered from within the treatment programme. See Table 2 for an overview of measurements and time points. Data is stored by Odense Patient data Exploratory Network. Data is managed by a local data manger with training and prior experience in datamangement. The datamanager quality checks data continuously. Data is collected, transferred, and stored securely electronically and is approved by the Danish Data Protection Agency (J.nr.: 14/26634 Reg. nr.: 2008-58-0035).

**Table 2:** Overview of measurements

Variable	Instrument	Items	Time (mins)	Screening Baseline	Weekly	3 months	6 months	12 months
Questions taken from participants								
Demographics		n/a	1	X				
Current treatment		n/a	2	X				
Diagnostic interview	M.I.N.I.	n/a	15	X				X
Depressive symptoms	QIDS-SR16	16	3	X	Item 12	X	X	X
	PHQ-9	9	2	X	X	X	X	X
Treatment preference		1	1	X				
Patient expectancy	CEQ	8	2	X				
Working alliance	WAI-SF	12	3			X		
Technology alliance	TAI-SR^a^	12	3			X		
Client satisfaction	CSQ-8	8	3			X		
Satisfaction with the online programme	SUS^a^	20	3			X		
Drop-out	Number of sessions completed - data extraction	n/a	0				X	
	Patients reason for drop-out. Questionnaire	3	2				X	
Questions taken from therapists								
Working alliance	WAI-SRT	12	3			X		
Satisfaction with the online programme	SUS^b^	20	3			X		

^a^Offered to condition receiving blended treatment only, ^b^Has to be completed once per therapist after completion of the first treatment

#### Primary outcome

The Patient Health Questionnaire-9 (PHQ-9) [30] will be used as primary outcome measurement to evaluate change in severity of depressive symptoms. It is a nine items questionnaire which can be used to screen and diagnose patients with depressive disorders. The nine items are each scored on a 0–3 point scale with a total score ranging from 0–27 with higher scores indicating more severe depression. The PHQ-9 has been shown to have good psychometric properties [32].

#### Secondary outcomes

The Quick Inventory of Depressive Symptomatology Self-Report (QIDS-16-SR) [33] is used in addition to the PHQ-9 because it is a promising questionnaire for assessing depressive symptoms especially in specialised mental health care, whereas the PHQ-9 is developed for use in primary care. The QIDS-16-SR is a questionnaire that screens for depressive symptoms and assesses depression severity. It consists of 16 items each scored on a 0–3 point scale and includes symptom domains of MDD based on DSM-IV. The QIDS-16-SR has shown good psychometric properties [33, 34].

The Client Satisfaction Questionnaire (CSQ-8) [35] is used to measure the patients’ satisfaction with the treatments. The questionnaire consists of eight items measured on a four point scale with total scores ranging from 8–32 and has shown good psychometric properties [36]. Clinician satisfaction with the treatment will be assessed by the short form of the Client Satisfaction Questionnaire including only the three core questions of the csq-8.

To measure the patients’ expectancy and judgement of credibility of the treatments the Credibility and Expectancy Questionnaire (CEQ) [37] is used. The CEQ is a two part questionnaire measuring credibility and expectancy respectively. Both factors have been shown to be stable across different populations, with high internal consistency within each factor [37].

To indicate whether a difference is seen in the therapeutic alliance the short version of the Working Alliance Inventory (WAI-SR) is used. WAI-SR is a 12 item self-report questionnaire with responses on a five point Likert scale ranging from 1 (never) to 5 (always) [38, 39]. The questionnaire covers three dimensions of working alliance: (1) therapeutic goals, (2) tasks, and (3) bond and the sub scales have demonstrated good internal consistencies [40]. Both the patient and the ten items therapist version of the questionnaire will be administered. The alliance between the patient and the programme will be assessed with the WAI Online Therapy (TAI)(Labpsitec, 2014).

Finally, to measure the patients’ perception of the usability of the software, the System Usability Scale (SUS) [41] is used. It is a simple ten item scale giving a global view of subjective assessments of usability of a technology system. All items are measured on a 5-point scale ranging from ‘strongly disagree’ to ‘strongly agree’. Total SUS scores have a range from 0–100. The questionnaire has been found to be reliable and robust [42].

## Sample size and statistical analyses

The primary outcome is analysed by regressing the three-month follow-up PHQ-9 data on the baseline PHQ-9 score and a treatment group indicator. We will use an ancova model for this purpose. Missing values are handled using a full-information maximum likelihood estimator. In case of dropout, we will use the last observed values in a supplementary intention-to-treat analysis. Missingness sensitivity will be assessed with a best-/worst-case comparison, where missing values are replaced with the minimum and maximum values observed at follow-up.

The primary hypothesis is that the blended care intervention will be no less effective than the TAU intervention in reducing depression symptoms. A sample size of 130 in two balanced groups will yield a power of at least 80% to detect standardised mean differences above 0.5 on a normally distributed variable. This equivalence interval is slightly narrower than other estimates of minimally clinically important differences [43].

In addition, we will compare the development of PHQ-9 in the two groups using the weekly measurements in a mixed effects model. We will model the individual trajectories as random slopes, and then let the treatment group predict the slopes. Significant slope differences between groups will indicate either faster or slower treatment effects in the blended care group. In case of non-normal data, we will use an appropriate measure of transformation or a generalised linear model. Variables with significant variations between groups at baseline are introduced as control variables.

Secondary outcomes will be analysed using t-tests or a rank sum test. Where appropriate, differences between baseline and follow-up scores are used as outcomes instead of only comparing the follow-up score.

## Discussion

The present study aims to investigate the clinical effectiveness of CBT delivered in a format in which face-to-face consultations are alternated with online self-help modules, since this could hold promise to combine advantages of Internet based guided self-help and traditional CBT while preserving the clinical effectiveness and broad applicability of traditional CBT.

It is a strength of the present study, that the treatment in the two conditions have similar therapeutic content given that both treatments are captured in one coherent treatment manual detailing each intervention. Consequently, the study will reveal a very direct comparison with a minimum of the variance explained by differences in therapeutic methods.

The study is limited, however, by the fact that even though it is situated in specialised mental health care, patients are recruited from a clinic offering self-referral. Consequently, the results may be hard to generalise with regards to future implementation.

The present study will contribute to the knowledge in the field by comparing, in a direct and concise manner, B-CBT to the well known and well investigated traditional face-to-face CBT, which in many settings is considered the golden standard of psychotherapeutic treatment of depression.
